# Socialization of Coping in Pediatric Oncology Settings: Theoretical Consideration on Parent–Child Connections in Posttraumatic Growth

**DOI:** 10.3389/fpsyg.2020.554325

**Published:** 2020-09-18

**Authors:** Veronika Koutná, Marek Blatný

**Affiliations:** Institute of Psychology, Czech Academy of Sciences, Brno, Czechia

**Keywords:** pediatric cancer, socialization of coping, posttraumatic growth (PTG), parent–child relationship, benefit finding

## Abstract

This theoretical article aims to summarize the results of studies relevant to parental influence on coping with childhood cancer and provide implications for future research focused on parent–child connections in posttraumatic growth (PTG) following childhood cancer. Parental influence on child coping described by the socialization of coping and socialization of emotions theories has already been studied in connection with posttraumatic stress, but the role of parents in the process of PTG in the child has not been clearly described yet. Several studies focused on PTG in childhood cancer survivors and their parents simultaneously, but only two studies explicitly included a parent–child connection in PTG in statistical analysis. Studies suggest that child PTG may be facilitated through parental coping advice supporting emotion expression and that parent–child connection in PTG may be mediated by the child’s subjective perception of the parents’ PTG. More research is needed to describe specific strategies proposed by parents and leading to child PTG and design tailored interventions for the use in the clinical care of childhood cancer survivors and their family.

## Introduction

Pediatric cancer can be considered a traumatic experience potentially leading to posttraumatic stress symptoms (PTSSs) or posttraumatic stress disorder (PTSD) in childhood cancer survivors and their parents. Posttraumatic stress disorder, as defined by the *Diagnostic and Statistical Manual of Mental Disorders, 5th Edition* (*DSM-V*), include symptoms of reexperiencing, avoidance, negative cognitions and mood, and increased arousal (American Psychiatric Association [APA], 2013) and have been extensively studied in pediatric cancer patients and their families. Results of studies assessing PTSSs/PTSD based on this classification suggested that the majority of survivors adapt well (Brown and Kupst, 2016), with the risk factors including central nervous system-related cancer diagnosis, female gender, reduced social support, and problematic family environment (for review, see Bruce, 2006). Recently, a growing number of studies indicated that both survivors and their parents may report positive psychological changes as well, as a consequence of their cancer experience referred to as posttraumatic growth (PTG; Duran, 2013). In childhood cancer survivors, PTG has been connected with shorter time since diagnosis and treatment completion, older age at diagnosis, higher levels of perceived life threat, optimism, and social support (for review, see Turner et al., 2018).

In addition to these factors associated with the characteristics of the child or the event, the child’s adjustment and ability to cope with adverse circumstances are also related to family environment and parent–child relationship. Better child adjustment is connected to greater family cohesion, support, expressiveness, and less conflict (Van Schoors et al., 2017). The role of parental factors has been studied in connection with child cancer–related PTSSs (e.g., Okado et al., 2014; Monti et al., 2017; Racine et al., 2018). However, our knowledge is limited when it comes to the parents’ ability to foster PTG in their child and to the association between parental PTG and the child’s PTG.

This article reviews literature relevant to the subject of the role of parental coping assistance in the context socialization of PTG in childhood cancer survivors. First, we introduce PTG in childhood cancer survivors and the importance of parents in the development of PTG in children. Then we describe coping strategies and discuss their link to PTG. The main focus of this article is to outline the possibilities of applying the socialization of coping and socialization of emotions theories in the research of PTG following pediatric cancer. In the final part, focusing on the research and clinical implication, we propose a way the study of parent–child connections in PTG can deepen our understanding of the successful adaptation of childhood cancer survivors and enhance the psychosocial care of pediatric oncology patients and their families.

## Posttraumatic Growth

### Definition of Posttraumatic Growth

Posttraumatic growth can be defined as “positive psychological changes experienced as a result of the struggle with traumatic or highly challenging life circumstances” (Tedeschi et al., 2018, p. 3). These positive changes usually occur in the domains of personal strength, new possibilities, relating to others, appreciation of life, and spiritual domain. In the view of Calhoun and Tedeschi, PTG occurs through cognitive processing called rumination. The term *rumination* is usually understood as not easily controlled repetitive thinking, but in the model of PTG, rumination can take two forms. Soon after a traumatic situation, rumination tends to be automatic and intrusive, but over time, it transforms into a more deliberate cognitive process aiming to understand the meaning of the experience and restore or rebuild the shattered assumptive world and core beliefs. Deliberate rumination resembles rather reflective processing referred to as reappraisal or reflection. The basis of the PTG model lies in the interplay of these cognitive processes with pretrauma characteristics of the person, his/her beliefs, goals, and narrative and sociocultural factors. A detailed description of the PTG model can be found in Calhoun and Tedeschi (2006).

Posttraumatic growth does not include normative developmental changes. Although these developmental changes may be similar, the key feature of positive changes characteristic for PTG is their association with the struggle with a traumatic experience (Tedeschi et al., 2018). Posttraumatic growth can occur in parallel with developmental changes. Developmental changes and the level of cognitive/social/emotional abilities of the individual represent the background against which the PTG takes place. Of course, these factors can also affect the domains in which PTG occurs (e.g., spiritual domain of PTG may require cognitive/emotional maturity, which cannot be assumed in young children).

These positive changes in the aftermath of trauma have also been described by several similar terms: in addition to PTG, one can find terms such as benefit-finding, stress-related growth, or growth following adversity. Some researchers distinguish between these terms (e.g., Park, 2009), but in this article in line with Helgeson et al. (2006), we refer to these terms as synonyms.

Posttraumatic growth can be understood as both, a process of dealing with an adverse situation (coping strategy) or an outcome of positive psychosocial adjustment (Tedeschi et al., 2018). In Tedeschi et al.’s (2018) point of view, the difference between PTG as a process and PTG as an outcome depends on time because they are sequential. Helgeson et al. (2006) suggest that PTG assessed early after a traumatic experience may reflect cognitive strategy used to reduce stress, whereas PTG assessed after a longer time-lapse may reflect actual positive change. This distinction is usually not explicitly stated by researchers but can be derived from the methods used to assess positive changes. The most commonly used methods typically include questionnaires such as the Posttraumatic Growth Inventory (Tedeschi and Calhoun, 1996) or Stress-Related Growth Inventory (Park et al., 1996). These questionnaires include items asking if changes in specific domains occurred as a result of traumatic experience. Sample items are “as a result of specific traumatic experience [e.g., cancer], I developed new interests” or “I have a greater sense of closeness with others.” These methods measure PTG as an outcome—they ask about specific positive changes/outcomes in various domains. Of course, there are other methods and qualitative approaches to assess PTG (for review, see Park and Lechner, 2006), but to the best of our knowledge, none of them directly assess PTG as a process—basically, all the methods ask whether and in what dimension positive changes occurred, not how they occurred. Posttraumatic growth as a process seems to be more closely linked with the research on coping strategies and related concepts.

### Posttraumatic Growth in Pediatric Oncology

The model of PTG described by Calhoun and Tedeschi has been empirically supported among adults. However, studies included in the review by Meyerson et al. (2011) indicate that the concept of PTG represents a relevant research topic in children and adolescents as well. The model of PTG in children and adolescents has been described by Kilmer (2006) and Meyerson et al. (2011). They also acknowledge the role of cognitive processing but put more emphasis on social factors and the parent–child relationship. These factors are included in the model of PTG in adults as well, but in children, the role of parents may be even more essential (Tedeschi et al., 2018). The way parents cope with stress may serve as a model for the evaluation of the severity of an adverse event and choice of coping strategies used by their child. At the same time, parents’ ability to provide appropriate care to the child may be influenced by their own distress (Kilmer, 2006). Thus, parents have already been acknowledged as an important element in the process of PTG in children, but besides these relatively general descriptions, little is known about specific parenting behaviors promoting PTG in the child.

Pediatric oncology represents an area, where PTG is often studied in both childhood patients and their parents. Posttraumatic growth in at least one domain has been reported by more than 80% of childhood cancer survivors and their parents (Barakat et al., 2006). Other authors (e.g., Gunst et al., 2016) reported even 94% of survivors experience at least one positive consequence of cancer. The main domains of positive changes in survivors and/or their families include meaning-making, appreciation of life, increased self-awareness, family closeness, psychological maturity, greater compassion and empathy, new values and priorities, spiritual growth, new strengths, and increased recognition of vulnerability and struggle (Parry and Chesler, 2005; Duran, 2013; Picoraro et al., 2014). Posttraumatic growth outcomes in childhood cancer survivors have been connected with higher age at diagnosis and shorter time interval from diagnosis or treatment completion, higher perceived life threat and intensity of treatment, and perceived social support (Barakat et al., 2006; Phipps et al., 2007; Turner-Sack et al., 2012; Tremolada et al., 2016). To better understand the processes underlying PTG outcomes in childhood cancer survivors, we need to look more closely at the strategies they use to cope with their experience.

## Coping

### Definition of Coping and Coping Strategies

There are many approaches to define coping as well as attempts to categorize particular coping strategies. The general coping framework has been described by Lazarus and Folkman (1984), who provided the most frequently cited definition of coping. They defined coping as “constantly changing cognitive and behavioral efforts to manage specific external and internal demands that are appraised as taxing or exceeding the resources of the person” (p. 141). This approach postulates that not all responses to stress can be considered coping. The term “coping” is reserved for such responses involving conscious and volitional effort to manage stress as opposed to automatic and unconscious responses to stress. Lazarus and Folkman (1984) also described two primary categories of coping strategies: emotion-focused and problem-focused strategies. *Emotion-focused* strategies aim to reduce unpleasant emotions induced by the stressor (e.g., avoiding the problem, wishful thinking, distraction, positive reappraisal). *Problem-focused* strategies focus on dissolving or altering the stressor (e.g., identifying and solving the problem/cause of stress, seeking information).

Since that time, many other more nuanced conceptualizations and categorizations emerged, including approaches focusing more directly on coping with stressful experiences in childhood and adolescence and developmental factors contributing to successful adaptation (e.g., Compas et al., 2001; Skinner and Zimmer-Gembeck, 2007; Garcia, 2010; Zimmer-Gembeck and Skinner, 2011). Compas et al. (2012, p. 4) provided a framework suitable for the study of a child coping with illness. They define coping as “a collection of purposeful, volitional efforts that are directed at the regulation of aspects of the self and the environment under stress.” The strategies children and adolescent use to cope with chronic illness have been evaluated from several points of view. Aldridge and Roesch (2007) in their meta-analysis of coping and adjustment in children with cancer summarized coping strategies into two major taxonomies: (1) emotion vs. problem-focused and (2) approach vs. avoidance strategies. Emotion vs. problem-focused strategies reflects the distinction described earlier in this section, whereas approach vs. avoidance (or engagement vs. disengagement) refers to strategies directed toward dealing with the stressor and strategies aiming to withdraw from it. Approach strategies include, for example, planning, seeking guidance, support, or information leading to problem solving, while avoidance strategies include, for example, denial, distancing, self-blame, and helplessness. These two taxonomies (emotion vs. problem-focused and approach vs. avoidance) are not mutually exclusive. Particular coping strategy can fit within both dimensions (e.g., threat minimization strategy can be seen as both avoidance and emotion-focused strategy). Brown and Kupst (2016) in their overview of coping with pediatric cancer summarized several taxonomies of coping strategies and suggested a more general classification of coping strategies: strategies affecting the stressor (problem-focused, approach, and primary control coping) and strategies in which an individual must adapt to the stressor (emotion-focused, secondary control coping). The majority of research on child and adolescent coping with chronic illness has been guided by the frameworks reflecting the distinctions between active/primary, accommodative/secondary, and avoidant/disengagement coping strategies (Compas et al., 2012).

Regardless of the specific classification of coping strategies, it is important to note that none of these coping strategies can be seen as universally adaptive/effective or maladaptive. The efficacy of a particular coping strategy depends on the specific context and interaction between the demands of the stressful event and the nature of a given coping strategy (Compas et al., 2012). A coping strategy that was found to be effective at one point in time/in a specific situation may prove to be maladaptive on another occasion. This context-specific nature of adaptability of particular coping strategies can be illustrated on the research of coping with childhood cancer and its link to PTG in survivors.

### Coping and PTG in Childhood Cancer Survivors

The association of PTG with specific coping strategies is not clear. Meyerson et al. (2011) in their systematic review of PTG in children and adolescents, including studies of childhood cancer survivors, reported active and positive cognitive reappraisal coping strategies (approach coping) to be positively related to PTG. Aldridge and Roesch (2007), in their review of coping and adjustment in children with cancer, failed to prove an influence of the approach, avoidant, and emotion-focused coping strategies on psychosocial adjustment. The overall adjustment was influenced only by problem-focused coping, and this association was small and negative. A more recent study of coping and adaptation in childhood cancer survivors found a connection of better psychosocial adjustment with less frequent use of avoidant coping strategies and positive association of PTG with acceptance coping strategies (Turner-Sack et al., 2012).

These inconsistencies illustrate the context-specific nature of (mal)adaptivity of coping strategies and may be explained by the role of time since diagnosis in the coping–adjustment relationship. Emotion-focused strategies soon after diagnosis were associated with better adjustment, but their positive influence weakened over time. On the other hand, approach or problem-focused coping strategies were associated with poor adjustment shortly after diagnosis, while in later stages of the treatment, these strategies became effective (Aldridge and Roesch, 2007). As suggested by Wenninger et al., 2013), avoidant coping strategies may be beneficial in the initial stages of diagnosis and treatment, but in the long-term point of view, avoidance may relate to higher distress.

The initial phase of treatment and diagnosis may be connected to different stressful situations than the phase of treatment completion and survivorship. The ability to adjust coping strategies according to the demands of a specific situation is called coping or regulatory flexibility (e.g., Bonanno and Burton, 2013; Cheng et al., 2014) and has already been linked to PTG in medical settings in adult patients with cancer (Pat-Horenczyk et al., 2016) and spinal cord injury (Kunz et al., 2018). Coping flexibility, as an ability to choose an appropriate coping strategy depending on the demands of a specific situation, appears to be more important than relying on a specific coping strategy or category of strategies. The “pool” of coping strategies available for the child to choose from may be shaped by a wide range of factors including social environment and parent–child relationship (Skinner and Zimmer-Gembeck, 2007).

### Parental Influence on Child Coping

The parent–child relationship is one of the most important resources moderating the effect of child exposition to adverse circumstances. Power (2004) reviewed the literature concerning parents’ role in child coping and specified pathways how parents may influence child exposure to a traumatic situation, his/her appraisal of this situation, and coping. He found parental warmth, support, acceptance, and family cohesion promote active or problem-solving coping strategies in children. Moreover, he found that parents who provide their child with opportunities to express emotions and help with understanding the event can thereby promote the ability to find meaning or something positive in the adverse experience. Specific practices of how parents can promote the development of coping in their child have been described in the framework of *socialization of coping* and *emotion socialization*.

#### Socialization of Coping

Kliewer et al. (1996) and Kliewer et al. (2006) in their socialization of coping model define three ways of parental influence on child coping and adjustment in middle childhood and adolescence: coaching, modeling, and context. Coaching refers to the direct suggestions parents give their children about the appraisal of threat and how to respond to it. Parents can influence their child’s ability to cope by guiding his/her perception of a situation as threatening or harmless and suggest specific actions to deal with it. Modeling represents the child’s observations of the parent’s own appraisals and their coping behaviors used to deal with the threat. As suggested by the social learning theory, children can learn how to handle the threat by observing what their parents do in similar conditions. Context refers to a more general pattern of family interactions and broader relational background in which parent–child interactions occur. These three ways of parental influences on child adjustment may be further affected by several broader factors including qualities of the child (e.g., age, gender), qualities and resources of the caregiver (e.g., personality, values), and family demographics (e.g., income, education).

Abaied and Rudolph (2010) described two models of the effect of socialization of coping in youth during the transition to adolescence: stress amplification-effects model and stress differential-effects model. The stress amplification-effects model proposes that the effect of coping socialization is negligible in youth with mild levels of stress because the level of stress does not exceed their coping capacities and resources. In a high level of stress, child coping capacities may not be sufficient for dealing with the situation individually, which makes them more dependent on parental coping guidance. In this case, the effect of socialization of coping is heightened. This model has been supported in the context of interpersonal stress. On the contrary, the differential-effects model predicts that youth are sensitive to parental coping advice regardless of the levels of stress, but the effect of coping advice may differ depending on the level/kind of stress (e.g., higher levels of engagement coping advice in case of a stressor of minor significance may be maladaptive). This model occurs in the non-interpersonal cause of stress, where different types of coping advice predict different outcomes depending on characteristics of the stressor but are equally significant in times of mild and high stress. Thus, this study implies the importance of contextual factors in determining the influence of parental coping advice on child adjustment.

#### Emotion Socialization

Coping with stressful situations is closely tied to emotional competence and the ability to understand and regulate emotions induced by the stressor, which can be developed through emotion socialization. Eisenberg et al. (1998a,b) described the model of parental socialization of emotion. Similarly to the socialization of coping model, emotion socialization theory proposes that parents may facilitate the development of child emotional competence by their reactions to children’s emotion, discussion of emotions, and their own expression of emotions. Parental supportive or non-supportive reactions to the child’s emotions, especially the negative ones, may help their child to maintain an optimal level of arousal or contribute to overarousal. By explicit discussions about emotions with the child, parents may express support and shape the child’s awareness and understanding of emotions. Last but not least, parental expressions of their own emotions may influence the child’s emotional competence in several ways—directly through processes of imitation/contagion or indirectly, for example, through mediating more general aspects of parenting.

Morris et al. (2007), in their Tripartite Model of the Impact of the Family on Children’s Emotion Regulation and Adjustment, described the development of children’s ability to regulate emotions in terms of resonating even more strongly with the model of socialization of coping. In their point of view, a child can learn to regulate emotions through observational learning and modeling, parenting practices (emotion coaching including parental reactions to emotions), and through the emotional climate of the family.

Emotion socialization is a dynamic process, which needs to be perceived in the developmental perspective. The effectivity of parental strategies aimed at helping their child regulate his/her emotions and cope successfully with adverse events depends on the age or developmental stage of the child. To be beneficial for a child adjustment, parental behaviors related to emotion socialization need to reflect increasing emotional competences and the need for the child’s autonomy. Parents’ emotion socialization practices, which can be considered beneficial in a particular developmental stage, may turn out to have a null or deleterious effect when used in different stages (Mirabile et al., 2018; Nelson and Boyer, 2018). It is also important to note that pathways of parental influence on the child are bidirectional. Parental practices can shape the child’s characteristics, behaviors, or outcomes, and vice versa, the child’s characteristics, behaviors, or outcomes can shape parental practices (e.g., Lengua and Kovacs, 2005).

Socialization of coping and emotion socialization theories describe parental influence on the child’s ability to handle adverse situations in general, but both provide important implications for the study of PTG in childhood cancer survivors as well.

### Parent–Child Associations in Coping With Pediatric Cancer

#### Socialization of Coping With Childhood Cancer

Both socialization of coping and socialization of emotion were utilized and/or empirically supported in childhood cancer patients. In this chapter, we report only the results of studies related to parent–child connections in coping with pediatric cancer. For more details about these studies, see Tables 1, 2. Table 1 presents the details about studies of parent–child associations in coping with cancer in general, whereas Table 2 is focused on the studies devoted to parent–child associations in PTG following pediatric cancer. Both tables present important additional information for understanding the results of mentioned studies with regard to the age range (development stage) of the sample, study design, and specific methods used to assess individual variables (especially coping, PTG and PTSD).

**TABLE 1:** Parent–child associations in coping with cancer.

**Study**	**Sample**	**Method**	**Results**
Barakat et al. (1997)	309 Childhood cancer survivors (50% female) aged 8–20 (mean = 13.53, SD = 3.37)	Children and parents: IES, Posttraumatic Stress Reaction Index, Assessment of Life Threat and Treatment Intensity Questionnaire	Higher levels of PTSSs for parents of children with cancer than comparison parents. Higher levels of PTSSs in parents compared to childhood cancer survivors. Parent–child associations in PTSSs in families with cancer. PTSSs in survivors and parents associated with perceived life threat and family and social support resources
	309 Mothers, 213 fathers	Only children: Revised Children’s Manifest Anxiety Scale, Trauma Symptom Checklist for Children	
	healthy parent–child dyads		
		Only parents: STAI, Family Adaptability and Cohesion Evaluation Scale, Social Network Reciprocity and Dimensionality Assessment Tool	
Colletti et al. (2008)	Parents (85.5% mothers) of 62 pediatric cancer patients (34 males) aged 2–12 (mean = 5.72, SD = 2.53)	Severity of Illness Scale, Parent Protection Scale, Child Vulnerability Scale, Parenting Stress Index/short form, Behavior Assessment System for Children–2nd Edition	Higher levels of parenting stress associated with poorer child behavioral, emotional and social adjustment. Higher levels of perceived child vulnerability associated with poorer emotional adjustment
		Social Skills Rating System (all only parent-report)	
Faith et al. (2019)	134 Pediatric cancer patients (36.8% female) aged 8–19 (mean = 12.99, SD = 3.14)	Parents’ Beliefs about Children’s Emotions Questionnaire	Differences in parents’ beliefs about patients’ and siblings’ emotions (patients perceived as more vulnerable). Patients’ maladaptive coping positively predicted by parents’ maladaptive and self-reliant coping and negatively predicted by parents’ support-seeking coping. Sibling coping not predicted by parental coping
		Brief COPE inventory (parents)	
	Parents (80.5% mothers), 124 nearest-age siblings	Self-report coping scale (patients/siblings)	
Hildenbrand et al. (2011)	15 Pediatric cancer patients (8 males) aged 6–12 (mean = 8.8, SD = 1.7)	Semistructured interview (child and parent version) questioning about stressors during cancer diagnosis and treatment and about coping: “What things do you do to (cope/help your child cope) or deal with (your/his or her) cancer/cancer treatment?”	Wide range of coping strategies reported across the sample but the number of strategies reported within families limited. Congruence between child-reported use of coping strategies and parent-reported coping assistance except for emotional expression (children reported using but parents did not report encouraging this strategy)
	15 Caregivers (73.3% mothers)		
Hildenbrand et al. (2014)	15 Pediatric cancer patients aged 6–12 (mean = 9.47, SD = 1.99)	Semistructured interview (child and parent version)	High congruence between child-reported use of coping strategies and parent-reported coping assistance
	17 Parents (59% mothers)	How I Coped Under Pressure Scale (patients)	
		Parent Socialization of Coping Questionnaire (parents)	
Landolt et al. (2003)	206 Pediatric patients (30 cancer patients) aged 6.5–14.5 (mean = 10.2, SD = 2.0 for cancer subgroup)	Child PTSD Reaction Index (interview with patients)	Child PTSSs not significantly related to parental PTSSs
		Posttraumatic Diagnostic Scale (parents)	
	Parents		
Monti et al. (2017)	166 Pediatric cancer patients (51.2% female) aged mean = 13.47, SD = 2.47)	T1: approx. 2 months after diagnosis or relapse: parents reported on their coping and depressive symptoms, children reported about their own coping	Fathers’ secondary control coping (T1) predicted secondary control coping in children (T2)
			Mothers’ depressive symptoms (T1) predicted lower levels of secondary control coping in girls (T2). Fathers’ depressive symptoms (T1) predicted lower levels of secondary control coping across sex (T2). Parental predictors of children’s coping not moderated by age
	161 Mothers and 83 fathers		
		T2: approx. 12 months later: children reported about their own coping	
		RSQ_PC (patients and parents)	
		BDI-II (parents)	
Murphy et al. (2017)	39 Adolescents with cancer (46% female) aged 10–15 (mean = 12.35, SD = 1.87)	T1: approx. 2 months after diagnosis: adolescents’ self-report: RSQ_PC, Self-Reported Positive and Negative Affect	Secondary control coping (e.g., cognitive appraisal) predicted higher levels of positive affect
			Maternal positive mood predicted adolescent positive mood (measured concurrently). Maternal negative affect not related to adolescent negative affect
	Mothers	T2: approx. 3 months later: video-recorded discussion of adolescents and mothers about experiencing positive and negative affect during cancer	
Nakajima-Yamaguchi et al. (2016)	33 Mothers, 1 father of a child with cancer aged 4–17 (mean = 10.5, SD = 3.9)	IES_R, Parent Experience of Child Illness, CBLC (only parent reports)	Parental PTSSs associated with child emotional and behavioral problems
Okado et al. (2014)	225 Dyads of parents and pediatric cancer patients (48.2% female) aged 8–17 (mean = 12.61, SD = 2.88), time since diagnosis: 3 months–17.42 years	Patients: CDI, UCLA PTSD, Screen for Child Anxiety Related Emotional Disorders, Life Events Scale	Cancer group: Parental depression and distress linked with child depression, anxiety and PTSSs. Parental PTSSs and anxiety related only to the same type of symptoms in the child
		Parents: BSI, IES_R	
			Control group: parental distress not related to child distress
	142 Healthy comparison dyads		
Okado et al. (2016)	225 Pediatric cancer patients (48.2% female) aged 8–17 (mean = 12.61, SD = 2.88)	Patients: CDI, UCLA PTSD, Screen for Child Anxiety Related Emotional Disorders	Parental anxiety, child depression, and parental and child PTSSs not related with time since diagnosis
		Parents: BSI, IES_R	Parental anxiety linked to child anxiety
			Parental PTSSs linked to child PTSSs
			Time since diagnosis moderated the associations between parental symptoms and child-reported anxiety and PTSSs: parent and child symptoms more strongly linked in families with longer time elapsed since diagnosis
	Time since diagnosis: 3 months–17.42 years		
	255 Parents		
Ozono et al. (2007)	88 Adolescent cancer survivors (55% female), mean age = 16.2 (SD = 2.3)	IES_R, STAI, Family Assessment Device, Life events data (Holmes–Rahe measure of social adjustment)	The prevalence of PTSSs higher for mothers and fathers than for survivors. Significant correlation of PTSSs between mothers and survivors. Higher trait anxiety and medical sequelae as predictors of PTSSs for survivors
	87 Mothers, 72 fathers		
Phipps et al. (2005)	162 Pediatric cancer patients in 4 groups differing in time since diagnosis, mean age = 15.9 (SD = 5.2)	Child-self report, parent-self and proxy reports: UCLA PTSD, IES_R	Low level of PTSSs among survivors in general. Higher levels of PTSSs in survivors with more recent diagnosis
			Concordance of survivors-self and parent-proxy reports. Parental and survivors-self reported PTSSs significantly correlated
	parents		
Robinson et al. (2007)	95 Pediatric cancer patients (35% female), mean age = 12.02 (SD = 2.51)	Patients: CDI, Roberts Apperception Test for Children	Associations between parent and child distress. Family environment and demographic characteristics (child age and gender) mediating parent–child associations in distress
		Parents: Symptom Checklist 90–Revised, Family Environment Scale, Norbeck Social Support Interview, CBCL	
	94 mothers, 67 fathers		
	Comparison families without pediatric chronic illness		
Schepers et al. (2019)	206 Childhood cancer survivors (49% female) aged 8–21 (mean = 15.04, SD = 3.22)	Survivors/peers: Parental Bonding Instrument, Behavior Assessment System for Children	Parent–child relationships mediate the link between parent functioning and youth adjustment for survivors as well as healthy peers
	Parents (87.9% mothers) 132 Healthy peers and parents	Parents: BSI, PTGI, Parenting Relationship Questionnaire	Parental distress impacts child adjustment more strongly than parental growth
Stuber et al. (1996)	64 Pediatric leukemia survivors (50% female) aged 7–19 (mean = 14.0, SD = 3.2)	Posttraumatic Stress Disorder Reaction Index (child and adult version)	Significant correlation between PTSSs in mothers and survivors, no association for PTSSs in fathers and survivors
	63 Mothers, 42 fathers		
Trask et al. (2003)	28 Pediatric cancer patients (57.1% female) aged 11–18 (mean = 13.61, SD = 1.87)	Patients: CBCL Youth-Self Report, Social Support Scale for Children, Family Adaptability and Cohesion Evaluation Scales-II, Coping Strategies Inventory	Parental distress positively associated with internalization symptoms in patients. Disengagement coping in parents related to disengagement coping in patients. Parents as key sources of social support
	Parents	Parents: BSI, Coping Strategies Inventory	

*CDI, Children’s Depression Inventory; UCLA PTSD, UCLA PTSD Reaction Index for *DSM-IV*; BDI, Beck Depression Inventory; PTGI, Posttraumatic Growth Inventory; CBCL, Child Behavior Checklist; IES(_R), Impact of Events Scale (revised); BSI, Brief Symptom Inventory; STAI, State-Trait Anxiety Inventory; RSQ_PC, Responses to Stress Questionnaire–Pediatric Cancer version.*

**TABLE 2:** Parent–child associations in positive adjustment and PTG.

**Study**	**Sample**	**Method**	**Results**
Howard Sharp et al. (2015)	153 Youth with cancer history (49.7% female) aged 8–19 (mean = 13.96, SD = 2.97)	Hemingway Measure of Adolescent Connectedness	Connectedness with parents may support the development of resilience. Connectedness of youth with cancer comparable with the comparison group. High level of connectedness associated with a low level of PTSSs and high level of benefit finding. Adolescents reported increased autonomy from family and stronger connectedness with the peer group
	Parents, 101 peers without chronic illness	UCLA PTSD (self and parent-proxy report), Benefit Finding/Burden Scale for Children, Life Events Scale	
Howard Sharp et al. (2016)	201 Youth with cancer history (50% female) aged 8–21 reporting on cancer vs. non-cancer event, mean age for the subgroup reporting on cancer event = 15.32, SD = 3.18	Emotions as a Child Scales-II, Benefit Finding subscale of the “Benefit/Burden Scale for Children”	Perceptions of parental reactions to youth’s distress linked to youth’s PTG. Youth reporting about cancer-related event perceived their parents as reacting with more support and reassurance/distraction compared to non-cancer event subgroup. Parental support and reassurance/distraction as possible mechanisms facilitating PTG
Koutná et al. (2017)	120 childhood cancer survivors (50.8% female), T1 mean age = 11.7, median = 10.7	T1 (1.7–7 years off treatment): Minneapolis-Manchester Quality of Life; subscale psychosocial functioning, Social and Health Assessment questionnaire: Parent–child interactions subscale	PTG and PTSSs in survivors are more affected by factors of parenting (parental warmth) and the emotionality of childhood cancer survivors than by objective medical data
		T2 (4–12.5 years off treatment): UCLA PTSD, BFSC	
Michel et al. (2009)	41 Childhood cancer survivors (53% female) aged 12–16 (mean = 13.7, SD = 1.1)	Survivors: BFSC, Kidscreen 27, UCLA PTSD, Youth Life Orientation Test, Brief Illness Perception Questionnaire	No association between children’s benefit finding and parents’ PTG. Benefit finding in children is associated with optimism, illness experience, younger age, and leukemia diagnosis
	45 Parents (89% mothers)	Parents: PTGI, SF-12v2, PTSD Checklist–Civilian Version, Brief Illness Perception Questionnaire	
Yaskowich (2002)	54 Pediatric cancer patients (35% female) aged 8–25 (mean = 16.1, SD = 3.36)	Child self and parent-proxy report: PTGI–Revised for Children and Adolescents, Social Support Scale for Children, Perceived Self-Efficacy–Revised	No significant correlations between children’s PTG and or parents’ PTG. No significant differences between parents’ and children’s reports of the child’s PTG. Children’s awareness of their parents’ PTG experience positively correlated with children’s PTG
	Parents		
		PTGI (parent-self report)	
		Semistructured interview: child PTG and awareness of parental PTG	

*PTGI, Posttraumatic Growth Inventory; BFSC, Benefit Finding Scale for Children; UCLA PTSD, UCLA PTSD Reaction Index for *DSM-IV*.*

Guided by the socialization of coping theory, Hildenbrand et al. (2011, 2014) analyzed parent–child connections in coping with childhood cancer. They found a high level of congruence between child self-reported coping strategies and parent-reported coping assistance (parental suggestions about children’s coping strategies). Similarly, Trask et al. (2003) reported a parent–child connection in increased use of disengagement coping strategies. Hildenbrand et al. (2011, 2014) also found that parents can facilitate the child’s adjustment to cancer through the support of approach-oriented coping strategies (i.e., cognitive restructuring and seeking social support).

Using the framework of emotion socialization, Howard Sharp et al. (2016) found that youth with cancer history perceive differences in their parents’ reaction to cancer- and non-cancer-related distress. Parents were perceived as more supportive and using more reassurance/distraction reactions in cancer-related stress compared to perceptions of youth reporting about non-cancer-related event. Faith et al. (2019) reported about the differences in beliefs parents hold about the socialization of emotion in their child with cancer compared to healthy sibling and about the different impact of parental coping on the sick and healthy child. Parents more strongly believed that all emotions (even the positive ones, if they’re too strong or intense) could have negative consequences for the child with cancer compared to healthy siblings. In this study, coping strategies used by parents predicted the use of maladaptive coping strategies in the child with cancer, but not the use of adaptive ones. Interestingly, parental coping was not related to healthy sibling coping.

Parental ability to provide coping assistance to the child may be affected by their well-being. High levels of parenting stress (stress in the parent–child system) predict poor behavioral and social adjustment of pediatric cancer patients (Colletti et al., 2008). Child adjustment is more strongly influenced by parental distress than by parental PTG (Schepers et al., 2019), and the parental–child association in adjustment to cancer tends to be stronger with a growing amount of time since diagnosis (Okado et al., 2016).

Parents overwhelmed with their own PTSSs may be less able to provide appropriate coping assistance to their child. These symptoms may then develop in the child as well. Several studies support the connection of parent’s own level of PTSSs with the level of PTSSs in their child in pediatric cancer settings (e.g., Barakat et al., 1997; Phipps et al., 2005; Robinson et al., 2007; Okado et al., 2016). Similarly, Nakajima-Yamaguchi et al. (2016) reported a connection of parental PTSSs with emotional and behavioral difficulties of their child with cancer. Stuber et al. (1996) found a significant correlation between PTSSs in survivors and their mothers, but not fathers. The mother–child connection of PTSSs was reported also by Ozono et al. (2007). Murphy et al. (2017) found that maternal positive emotionality predicts positive emotionality and lower levels of PTSSs in the child. The effect of parental well-being on child coping with cancer has been empirically supported by Monti et al. (2017) as well. This study suggests positive child adjustment to cancer may be promoted through interventions targeting parent’s own psychosocial functioning, especially their own coping and depressive symptoms. Moreover, Monti et al. (2017) found that the parent–child association in coping may be time-specific. Mother–child association in coping emerges earlier with more immediate effect, whereas father–child association emerges later with the effect unfolding over time.

In our point of view, parent–child connections in posttraumatic stress following childhood cancer correspond with all three ways of parental influence on child coping proposed by the socialization of coping theory. Parents with elevated symptoms of PTS may provide their child with maladaptive coping advice, serve as models for adopting ineffective coping or emotion regulation strategies, and create a problematic background for dealing with adverse events. However, the evidence connecting parental functioning with child adjustment is not conclusive. For example, Landolt et al. (2003) failed to find a parent–child connection of PTSSs. Although the majority of studies found that a parent–child connection plays a role in the adjustment to cancer, it should be noted that most of these studies employed a cross-sectional design, which does not make it possible to show the direction of the influence.

#### Parent–Child Association in Positive Adjustment and PTG Following Pediatric Cancer

As the previous section indicates, there is some evidence that the parent–child connection plays a role in PTSSs following childhood cancer. Higher levels of PTSSs in parents (especially mothers) appear to be connected with higher levels of PTSSs in their child. There may be two possible reasons for this correlation: (a) the parents’ reduced ability to provide the appropriate care and support to their child due to their own PTSSs and (b) the socialization of maladaptive coping and emotion regulation strategies used by the parents. If these are the ways by which parents can moderate child adaptation in terms of PTSSs, parental warmth and socialization of specific coping or emotion regulation strategies could promote positive adjustment and PTG.

The parent–child relationship is supposed to be one of the key elements of the PTG model in children (Kilmer, 2006). In this model, factors of the family environment are represented mainly by parental posttrauma responsiveness and factors associated with relationships and available support. The ability of parents to cope successfully and provide their child with a safe haven and support in dealing with the traumatic event promotes the occurrence of PTG in their child (Hafstad et al., 2010). Kilmer and Gil-Rivas (2010) found that positive reappraisal coping advice has been positively associated with child PTG. Kilmer et al. (2014) explicitly proposed caregiver PTG and parental coping guidance as a factor related to child posttrauma functioning and deliberate rumination leading to PTG. In their point of view, the way parents communicate with their children about the stressful experience may affect child’s appraisal of the event and subsequent coping strategies. Parental contributions to child PTG have also been discussed in the study of bereaved youth by Wolchik et al. (2008), who found a connection of seeking support from surviving parent or “guardian” and PTG. They suggested that parents may enhance cognitive processing leading to PTG in their child by providing opportunities for the disclosure and validation of emotions, experience of acceptance, and suggestions of new posttrauma schemas.

In the specific context of pediatric cancer, Howard Sharp et al. (2015) found that connectedness with parents as well as peers may serve as a possible mechanism facilitating PTG in children with cancer. Howard Sharp et al. (2016) found that the diagnosis of pediatric cancer does not decrease parental ability to provide appropriate emotional-related parenting behavior. It is more of a shared stressor for the family and parental support during the process of dealing with this experience can promote PTG in the child. Koutná et al. (2017) analyzed predictors of PTSSs and PTG in childhood cancer survivors and found that both, PTG and PTSSs, are more strongly affected by factors of parenting and emotionality of childhood cancer survivors than by objective medical data. In line with studies of the parent–child relationship and protective factors for the risk of symptoms of poor psychosocial adaptation, the results of this study suggest that warm parent–child interactions may protect survivors from PTSSs and facilitate the process of finding benefits in childhood cancer survivors.

In contrast with PTSSs literature, there are only a limited number of studies directly considering parent–child associations in PTG following pediatric cancer. Michel et al. (2009) addressed this issue and found no relationship between child benefit finding and parental PTG. Yaskowich (2002) found child awareness of parental PTG was positively related to their own PTG, while parent- and child self-reported PTG scores were unrelated. This suggests that child subjective perception of their parents’ PTG may be more important than PTG reported by parents. It is not clear whether child perception of positive changes in parents contributes to their own PTG or vice versa. However, it suggests that parental PTG, or at least its subjective perception by the child, may represent an important element in the process of PTG in the child. Apart from the parent–child connection in PTG, Berger and Weiss (2009) suggest a PTG expansion to the whole family system going beyond PTG associations of individual family members, but their clinically guided approach still awaits empirical support.

## Implications for Future Research

Posttraumatic growth in survivors has been connected mainly to the factors related to child or event characteristics (e.g., age, gender, optimism, time since trauma, and subjective perception of its severity). Research failed to find conclusive evidence connecting particular coping strategies with positive psychosocial adjustment or PTG of childhood cancer survivors, because this connection may be mediated by time or specific context. Studies of PTG in adult population show that coping flexibility (adjusting behavior to the demands of a specific situation) is more beneficial than universal use of any single strategy, but more research is needed to verify the effectiveness of coping flexibility in childhood cancer survivors.

A child’s ability to cope is shaped by several influences, with parental guidance being one of the most important. The socialization of coping and emotion socialization theory offers a framework for the study of parental influence on child adjustment to adverse events. The pathways specified by these theories (coaching, modeling, and context) suggest that parents may actively engage in aiming their child toward the perception of positive consequences of adverse events and/or that child PTG may be affected by the parent’s own coping abilities and thus also by their own PTG. Although the parent–child associations in adjustment following adverse events are definitely not new in PTSSs literature, in case of PTG, up to now not much attention has been paid to the parent–child association. Several studies focused on PTG in childhood cancer survivors and their parents simultaneously, but only two studies (Yaskowich, 2002; Michel et al., 2009) explicitly included a parent–child connection in PTG in their statistical analysis. Some studies focused on the effect of context in terms of the family environment such as family cohesion, expressiveness, support or parental warmth, and its contributions to child PTG. Although we already know parenting styles in parents of pediatric oncology patients may be specific (Ernst et al., 2019), studies examining the other two ways of parental coping guidance, coaching (direct suggestions) and modeling (observational learning), are needed.

Parental coping assistance is largely determined by beliefs parents have about their child’s emotions and abilities. Beitra et al. (2018) refined the method grounded in the socialization of emotions theory, which has been widely used for the assessment of parental beliefs about shaping and development of child emotions among healthy children (Parent’s Beliefs About Children’s Emotions questionnaire, PBACE; for more details about this method, see Stelter and Halberstadt, 2011; Halberstadt et al., 2013). They created a shortened version with beliefs most relevant to parents of pediatric oncology patients and with reduced participant burden for the use in pediatric oncology. Future research incorporating the socialization of coping/emotion socialization perspective and methods could contribute to the clarification of the PTG process in children. The topic of parent–child connections in perceiving positive life changes following pediatric cancer as well as possibilities of PTG facilitation through parental coping guidance represents an underexplored research area. A more comprehensive study of parent–child connections in PTG following pediatric cancer could also enrich the limitedly explored concept of vicarious or secondary PTG, a transmission of positive changes to significant others (Arnedo and Casellas-Grau, 2014).

The study of parent–child connections in PTG offers important clinical implications as well. Providing psychosocial support to childhood cancer survivors and their parents throughout the cancer trajectory is one of the recommendations in Standards of Psychosocial Care of Children With Cancer and Their Families (Wiener et al., 2015). However, the authors participating in the development of these Standards agree that evidence-based interventions for psychosocial care focused on a family affected by childhood cancer are not widely available (Kearney et al., 2015). However, some already do exist. For example, there is the Surviving Cancer Competently Intervention Program (Kazak et al., 1999), a cognitive-behavioral family therapy aiming to reduce psychological distress in childhood cancer survivors and their parents/families. There are also programs focused on improving emotion socialization practices of parents in the general population such as Tuning in to Kids/Teens (Havighurst and Harley, 2010; Havighurst et al., 2012). Results of studies of parent–child connections in PTG and parental coping guidance promoting PTG in children could help refine or adapt tailored interventions for the use in the clinical care of childhood cancer survivors and their family to foster adaptation in the aftermath of cancer, but also in other types of adverse events (at least the medical ones).

## Conclusion

The available literature provides convincing support for the effect of protective factors connected to the parent–child relationship on the risk of maladjustment of a child struggling with an adverse event in general, or in the specific context of pediatric cancer. However, the role of this relationship in promoting PTG is yet to be understood. Studies suggest that parental coping advice supporting emotion expression may facilitate child PTG, but more research is needed to describe specific strategies proposed by parents and leading to child PTG because there is no conclusive evidence connecting specific coping strategies with child PTG. The relationship of specific coping strategies to overall child adjustment is further moderated by the time frame of the assessment. Socialization of coping and socialization of emotions theories represent a suitable framework for designing such studies. To date, there is only limited knowledge about the connection of parent’s and child’s own PTG. Available results suggest that this connection may not be straightforward but may be mediated by the child’s subjective perception/opinion about the parents’ PTG.

## Author Contributions

VK conducted the review and prepared the manuscript. MB supervised and revised the manuscript. Both authors contributed to the article and approved the submitted version.

## Conflict of Interest

The authors declare that the research was conducted in the absence of any commercial or financial relationships that could be construed as a potential conflict of interest.
